# Hair stress hormones in individuals with a recent suicide attempt experiencing a depressive episode

**DOI:** 10.1016/j.ynstr.2025.100776

**Published:** 2025-12-02

**Authors:** Lejla Colic, Anna Karoline Seiffert, Lydia Bahlmann, Ani Zerekidze, Johanna Walther, Larissa McClain, Bianca Besteher, Fabricio Pereira, Mocrane Abbar, Martin Walter, Fabrice Jollant, Gerd Wagner

**Affiliations:** a Department of Psychiatry and Psychotherapy, Jena University Hospital, Jena, Germany; b German Center for Mental Health, Partner Site Halle-Jena-Magdeburg, Germany; c Department of Clinical Research and Innovation (DRCI), CHU Nîmes, Nîmes, France; d Mathématiques, Informatique, Physique et Application, Département Sciences et Arts, Université de Nîmes, Nîmes, France; e Department of Psychiatry, CHU Nîmes, Nîmes, France; f Center for Intervention and Research on Adaptive and Maladaptive Brain Circuits Underlying Mental Health (C-I-R-C), Site Halle-Jena-Magdeburg, Germany; g Faculty of Medicine, Paris-Saclay University, Le Kremlin-Bicêtre, France; h Department of Psychiatry and Addictology, Paul Brousse Hospital, APHP, Villejuif, France; i Department of Psychiatry, McGill University, Montreal, (Québec), Canada

**Keywords:** Suicide, Attempted, Hydrocortisone (cortisol), Corticosterone, Dehydroepiandrosterone, Hair analysis, Depressive disorder, Major

## Abstract

**Background:**

Stressful events and dysregulation of the hypothalamic-pituitary-adrenal (HPA)- axis contribute to the risk of suicide attempt (SA) in persons in depressive episodes. Hair cortisol, cortisone and dehydroepiandrosterone (DHEA) concentrations may serve as reliable indicators of HPA axis dysregulation prior to SA.

**Methods:**

Participants (n = 75; mean age [standard deviation] = 30.0 [10.2] years; n = 49 [65 %] women; Jena site) comprised three groups: individuals with a history of SA approximately one month (SA; n = 22); individuals with a current depressive episode without SA history (CC; n = 31) and healthy individuals (HC; n = 22). SA was defined as self-initiated, potentially injurious behavior accompanied by some intent to die. Stress hormones were measured using LC-MS/MS protocol (days from sampling to analysis = 342 [171]) and logarithmic transformed. Group differences in hair stress hormones with hair segments representing time were tested using linear mixed models on a p < .05 threshold. Exploratory models further examined the effects of childhood abuse, frequency of SAs, suicide intent level and impulsiveness of the last SA of mean hormone levels, on a corrected p_corr_< .012 threshold.

**Results:**

There was a main effect of group for the DHEA-log (p = .02) and post-hoc tests indicated that SA group had higher DHEA compared to CC (p_corr_ = .01) and HC (p_corr_ = .08) in the peri-suicidal period. There were no significant (p < .05) interaction or group effects on cortisol-log and cortisone-log. Preliminary exploratory analyses showed that SA with multiple attempts had higher mean DHEA-log compared to SA with a single suicide attempt (p_uncorr_ = .05). Furthermore, there were positive associations between level of suicide intent and both mean cortisol-log (p_uncorr_ = .02) and mean cortisone-log (p_uncorr_ = .02). Childhood abuse and impulsiveness of the last SA were not related to hair stress hormones.

**Conclusions:**

Individuals with a recent history of SA showed alterations in the DHEA hair levels. These results partially support dysregulation of the HPA axis as a biopsychosocial feature of SA. Future longitudinal and experimental studies should investigate whether hair HPA axis hormones can serve as markers of suicidal crisis and vulnerability.

## Highlights

• Suicide attempts (SA) may be connected to dysregulated stress response.
• Individuals with SA had overall higher hair DHEA.
• Individuals with multiple compared to single attempts had higher DHEA.
• Higher suicide intent was positively related to higher cortisol and cortisone.
• Childhood abuse and impulsiveness were not related to hair hormones.

## 1 Introduction

The World Health Organization (WHO) estimates that around 700 000 people annually die by suicide (World Health, 2025) and 10 to 20 times more people attempt suicide. Thus, suicide and suicide attempts (SA) are a serious global health problem (Turecki et al., 2019).

Most current models of suicide and SA, e.g., the diathesis-stress model (Mann and Rizk, 2020), hypothesize that these behaviors results from the complex interplay between a specific diathesis or “capability” and between triggering factors, such as acute and/or chronic stress including e.g. interpersonal conflicts and/or a mental disorder. Although mental disorders, like Major Depressive Disorder (MDD), are associated with increased suicide risk (Xu et al., 2023), they lack specificity for predicting suicidal behavior (Franklin et al., 2017). Because only a few patients with mental disorders die by suicide or attempt it, identifying those at highest risk is crucial. Furthermore, current research in suicidology recognizes that the development of suicide ideation and the transition from suicide ideation to behavior are distinct phenomena with distinguishable diathesis and risk factors (Franklin et al., 2017; Klonsky et al., 2016, 2017).

The Integrated Motivational-Volitional Model (O'Connor and Kirtley, 2018), a theoretical ideation-to-action model of suicidal behavior, conceptualizes the suicidal process as comprising three sequential phases: pre-motivational, motivational, and volitional. The pre-motivational phase, grounded in the diathesis-stress model, involves the interaction between the distal vulnerability factors, such as neurobiological alterations (e.g., serotonergic dysregulation or hypothalamic-pituitary-adrenal (HPA) axis dysfunction (Pompili et al., 2010) or behavioral components (e.g., impaired decision-making (Jollant et al., 2005) and proximal environmental stressors (e.g., trauma or significant life events (March et al., 2014). Socio-environmental factors, including socioeconomic disadvantage and unemployment, also serve as significant vulnerability markers (Iemmi et al., 2016). Furthermore, childhood abuse constitutes a further significant risk factor for suicide attempts (J＀＀J. Liu et al., 2017), with empirical evidence linking it to epigenetic modifications (Turecki, 2018) and altered cortisol regulation (Carpenter et al., 2011) during critical developmental periods. The motivational phase centers on psychological experiences of defeat and humiliation, often precipitated by interpersonal loss, social rejection, or socioeconomic status loss, all of which may lead to a feeling of entrapment (Li et al., 2018; Taylor et al., 2011). This entrapment reflects a state in which the individual perceives no viable means of escape other than suicide. The transition from defeat to suicidal ideation is influenced by cognitive-emotional moderators, such as hopelessness (Ribeiro et al., 2018) and deficits in coping and problem-solving abilities (Ambrus et al., 2020). The final volitional phase addresses the transition from suicidal ideation to behavior and is influenced by volitional moderators, including acquired capability for suicide (e.g., through habituation to pain or previous self-injury) (Smith et al., 2010), access to means (Lin et al., 2021), planning and impulsivity (R. TR. T. Liu et al., 2017). This phase also includes broader facilitating factors that are physiological, psychological, social, or environmental in nature and that contribute to behavioral enactment. A history of prior suicide attempts is a clinically important predictor within this phase (Ribeiro et al., 2016). Across all three phases, dysregulated HPA axis may exacerbate underlying vulnerabilities, intensify entrapment-related feelings and cognitions, and increase impulsivity, thereby contributing to the emergence of a suicidal crisis, particularly in the context of acute stressors such as a depressive episode or recent life adversity (Berardelli et al., 2020).

Chronic stress exposure induces lasting alterations in HPA axis function, affecting both basal glucocorticoid regulation and stress reactivity through distinct mechanisms (Herman et al., 2016). These effects vary by stressor type, severity, and individual factors such as physiological state (e.g., inflammation), coping capacity and genetic vulnerability (Miller et al., 2007; Russell and Lightman, 2019). Chronic stress exposure can result in either a HPA axis hyperactivation (e.g., elevated basal glucocorticoid levels) or a hypoactivation (e.g., blunted glucocorticoid response to an acute stressor) (Miller et al., 2007). These patterns highlight the HPA axis's complex and adaptive role in chronic stress (Chrousos, 2000; Herman, 2013). Cortisol and cortisone concentrations and release are regulated via a negative feedback loop through glucocorticoid receptors in the brain and pituitary gland (Spencer and Deak, 2017; Young et al., 2004). During HPA axis activation, dehydroepiandrosterone (DHEA), an anabolic steroid with neuroprotective and regenerative properties, is co-released with cortisol (Morgan et al., 2004). DHEA secretion in response to acute stress was thus suggested to exert a protective role by antagonizing the effects of other stress hormones (Dutheil et al., 2021).

While dysregulation of the HPA axis is frequently associated with various mental disorders (Malisiova et al., 2021; Wingenfeld and Wolf, 2011), it remains unclear whether HPA axis alterations observed in individuals with SA are distinct from those linked to psychiatric conditions more broadly. As such, the utility of HPA axis dysfunction as a specific predictive biomarker for suicidal behavior, independent of underlying mental illness, has yet to be conclusively established. In the context of the history of SA, research thus far has focused on investigating cortisol as the putative marker of dysregulation of the HPA-axis, mostly in individuals with mood disorders.

Alterations in the stress hormone release and concentrations can be investigated via resting or diurnal (basal) secretion, cumulative and long-term secretion, dexamethasone suppression test or reactive secretion after an acute physical or psychosocial stressor, and can be measured in saliva, blood, urine and hair (King and Hegadoren, 2002). Most studies investigating the link between SA and the HPA axis have focused on examining salivary and blood cortisol levels as basal or reactive secretion.

Studies examining baseline cortisol levels in blood or saliva have reported associations between a history of SA and decreased (Keilp et al., 2016), increased (Chatzittofis et al., 2013) or unchanged cortisol levels (Karlović et al., 2012; Strumila et al., 2024). A meta-analysis on this methodological approach indicated that overall there are no differences among those with a SA history compared those without; however, when age was taken into account, younger individuals showed increased, indicating hyperactivity, while older adults decreased cortisol levels, indicating hypoactivity of the HPA axis (O'Connor et al., 2016). Another meta-analysis indicated that methodological choices, such as the timing of sample collection or the comparison group used, also affect results (Hernández-Díaz et al., 2020). Similar to single resting measurements, studies examining baseline cortisol profiles, such as cortisol awakening response (CAR), have reported increased, blunted (O'Connor et al., 2020) or unchanged levels (Wiebenga et al., 2022) in persons with a history of SA.

Studies investigating cortisol reactivity to acute laboratory stressors have shown varied results, with findings indicating both elevated reactivity (Law et al., 2024) and blunted reactivity (Melhem et al., 2016; O'Connor et al., 2017). The heterogeneity in cortisol reactivity may be in part explained by moderating factors, such as suicide intent or impulsiveness of a suicide attempt. For example, suicide attempters characterized by high suicide intent exhibited attenuated stress-reactive cortisol and lower total cortisol output during stress-inducing task compared to attempters with low suicide intent (Herzog et al., 2023). This study suggested that there are at least two subgroups of SA who may react differently to acute stressors. However, no clinical control group was investigated, making it difficult to evaluate the specificity of this finding. In connection, Bernanke et al. (2017) proposed an interesting framework of two SA subtypes characterized by high versus low reactivity to stressors. The authors postulated that individuals characterized by a stress-reactive pattern show a stronger HPA axis reactivity and exhibit more impulsive SA.

The dexamethasone suppression test, another method to assess HPA axis function, showed in a recent meta-analysis, that non-suppression, indicating HPA axis alterations, are slightly to moderately associated with death by suicide but not with past SA (Spaan et al., 2024), suggesting distinct psycho-biological profiles. The analysis also identified moderating variables, such as the type of attempt (violent vs. non-violent) and the level of suicidal intent, contributing to varied results.

Heterogeneity in reported results may be in part due to methodological constraints of single day measurements of samples from blood, saliva or urine. In contrast, measuring stress hormones in hair is a promising method for capturing long-term HPA axis activity, as it reflects cumulative hormone concentrations over a period of 1–3 months (Russell et al., 2012). Compared to single-point measurements, hair analysis may provide a more accurate indicator of chronic HPA axis dysregulation and is less susceptible to common confounding factors such as circadian variability, acute stress, or hormonal influences from contraceptive use. Moreover, it was suggested that hair glucocorticoid relates to the overall output, *i.e.*, post-awakening and mean diurnal variation (Greff et al., 2019; Short et al., 2016; Stalder et al., 2017). Thus, hair stress hormone levels may indicate global changes in the HPA axis activity and chronic stress levels (Kalliokoski et al., 2019), potentially preceding and around suicidal crisis. While promising, to our knowledge only few studies investigated hair stress hormones, e.g., cortisol in individuals with SA (Melhem et al., 2017). reported lower hair cortisol in young individuals (ages 15–30) and who attempted suicide, which was also related to higher self-reported childhood abuse and perceived stress. Adults with recent SA (past month) and depression showed lower hair cortisol levels in two consecutive hair segments compared to those with depression alone and healthy controls (Jiang et al., 2025). On the contrary (Karabatsiakis et al., 2022), reported higher levels of hair cortisol in older individuals (ages >40) who died by suicide compared to healthy controls and individuals with major depressive disorder (MDD), while hair cortisol was related to depression severity. Similar to findings from the meta-analysis on dexamethasone suppression test, these three studies suggest HPA axis differences with hypoactivity in persons with SA history and hyperactivity in those who died by suicide.

To expand aforementioned studies, our primary aim was to investigate putative markers of altered HPA axis activity in individuals following a recent suicide attempt. To control for the potential confounding effects of comorbid mood disorders and assess the specificity of our findings, we included a control group of depressed individuals with no history of suicide attempts. Hair cortisol, cortisone, and DHEA were measured across two segments: one reflecting the period surrounding the suicide attempt (approximately one month prior to hair collection), and another reflecting the approximately two months prior. This design allowed us to explore potential longer-term alterations in HPA axis activity and cumulative exposure to chronic stress.

For the primary aim and based on previous findings from hair cortisol in SA (Jiang et al., 2025; Melhem et al., 2017), we hypothesized that individuals with a recent SA would exhibit HPA axis hypoactivity in the period around the attempt, as reflected by lower levels of hair glucocorticoids, cortisol and cortisone compared to both the healthy and clinical control groups. Based on the research that connects chronic stress with lower levels of baseline DHEA in hair (Qiao et al., 2017) and saliva (Heany et al., 2018) we hypothesized that the SA group would also have lower levels of hair DHEA levels in the month around the attempt. Both lower glucocorticoids and DHEA levels in the peri-suicidal time (as compared to the time before) would indicate maladaptive response of the HPA axis to the stressful events surrounding SA, i.e., they would indicate adrenal fatigue (Kamin and Kertes, 2017).

For the secondary preliminary analysis, we investigated potential moderators (childhood abuse, number of previous SA, severity of suicide intent and impulsiveness of the last attempt) on the HPA axis in the SA group. To investigate these associations, we carried out four exploratory secondary analyses, corrected for multiple testing, using mean stress hormone concentrations across the two hair segments. We hypothesized that persons with higher childhood abuse will show lower levels of glucocorticoids (O'Connor et al., 2018). Moreover, individuals with multiple attempts compared to those with a single attempt are known to differ on clinical and psychological variables, for example higher severity of psychopathology, motor impulsiveness, and self-reported severity of childhood abuse (Colborn et al., 2017; Lübbert et al., 2021). The occurrence of multiple attempts may indicate more frequent stressful periods and/or a higher sensitivity to stress resulting in lower levels of glucocorticoids and DHEA (adrenal exhaustion). Based on previous research, we hypothesized that individuals with lower suicide intent or those with impulsive suicide attempt may demonstrate greater stress sensitivity (Bernanke et al., 2017) reflected via higher levels of glucocorticoids.

## 2 Methods

### 2.1 Participants

This study was part of a project: “The choice of a violent suicidal means: a MRI study with computational modeling of decision-making” (grant number LSRG-1-086-19). The project was done in the Department of Psychiatry and Psychotherapy, at the Jena University Hospital, Germany, and the Department of Psychiatry at the Nîmes Academic Hospital (CHU), France. The Friedrich Schiller University, Jena, Germany, and the *Comité de Protection des Personnes SUD-EST IV*, France approved this project. The study was registered on ClinicalTrials.gov (NCT05230043).

Three groups were recruited: persons with recent history of SA (SA group); persons with MDD or a BD but no personal history of SA (clinical control (CC) group); and healthy controls with no history of SA or MDD/BD (HC group).

Inclusion criteria for all participants were ages 18–60 years, both genders and the ability to speak German or French fluently. Inclusion criteria for the SA group were based on the Diagnostic and Statistical Manual of Mental Disorders (DSM)-5 (APA, 2013) criteria for *current* suicide behavior disorder: “A self-initiated sequence of behaviors by an individual who, at the time of initiation, expected that the set of actions would lead to his or her death.” History and details of SA were determined using the Columbia Suicide Severity Rating Scale (C-SSRS; Posner et al., 2011) and a structured clinical interview. From the participants in the SA group (n = 24) twenty-two participants had their last SA < 39 days. Exclusion criteria for the SA group were loss of consciousness following suicidal act. Individuals in the CC group were included if they had MDD or BD and were in a *current* depressive episode. Exclusion criteria for the CC group included a personal or family history (up to 2nd degree) of SA, whether *current* or *past*, or death by suicide. Participants in the HC group were included if they had no *current* or *past* personal or family (up to 2nd degree) history of major psychiatric disorder or of SA/death by suicide. The presence or absence of diagnoses was established using the M.I.N.I International Neuropsychiatric Interview (M.I.N.I. 7.0) following DSM-5 criteria (Sheehan et al., 1998) by trained psychologist with a Master degree. The SA group consisted predominantly of individuals with MDD as a primary diagnosis (95 %), while the CC group consisted only of individuals with MDD as a primary diagnosis.

Other exclusion criteria for all groups were IQ below 80, current (hypo)manic symptoms, past brain trauma, neurological or inflammatory diseases (e.g., multiple sclerosis) and contraindications for magnetic resonance imaging. Only participants with natural (non-dyed) hair were included in the hair sampling part of the study.

A total of 131 participants were included in the overall study (92 in Jena, Germany, and 39 in Nîmes, France), comprising 49 individuals with SA, 34 clinical controls, and 49 healthy controls. Hair samples were unavailable for 56 participants due to insufficient sample mass, primarily at the Nîmes site, as well as hair dyeing, scalp visibility or refusal to provide a hair sample. The analysis was thus restricted to participants from the Jena site.

Final sample of this study comprised 22 participants in the SA group (mean age [standard deviation (SD)] = 29.5 [10.2] years, 16 women, 73 %), 31 participants in the CC group (30.0 [11.5] years, 21 women, 68 %) and 22 participants in the HC group (30.5 [8.6] years, 12 women, 54 %).

### 2.2 Hair stress hormones assessment

For all three groups hair, samples were collected on the experimental day in the Department of Psychiatry and Psychotherapy at the Jena University Hospital. For the SA group, hair was collected approximately within one month after their last SA (minimum-maximum = 4–38 days) and two participants that had >51 days from their last SA were excluded from the sample.

Two segments of hair were sampled following a standardized procedure of the Dresden Lab Service GmbH. We marked the segment one as the segment more proximal to scalp which is denoting peri-suicidal period (i.e. time around the last SA). Hair samples were collected between July 2021 and January 2023 and were stored in aluminum foil pouches in dry and dark place (cupboard) at room temperature.

Samples were shipped in the end of January 2023 to Dresden Lab Service GmbH where they were analyzed in February 2023 using the LC-MS/MS protocol described in (Gao et al., 2013) (days from the sampling to analysis, minimum-maximum = 21–569 days). In short, cortisol, cortisone and DHEA were extracted from the non-pulverized hair sample using methanol incubation. Column switching strategy for the on-line solid phase extraction was done, followed by the analyte detection on an AB Sciex API 5000 QTrap mass spectrometer. One participant (CC group) did not have hair long enough to provide segment two; 5 participants (1 in SA and 4 in HC group) for segment one and 11 participants (4 in SA, 1 in CC and 6 in HC group) for segment two had hair mass <3 mg and were excluded from the analysis. The lower detection value for cortisol and cortisone was .3 pg/mg and from those with hair mass >2.99 mg one participant (CC group) was excluded for cortisol for segment one. All samples were measured in a single batch. The intra-assay coefficient of variance was 9 % for cortisol, 6 % for cortisone and 11.8 % for DHEA.

### 2.3 Psychometric assessment

Depression severity were measured with the clinician reported Montgomery-Åsberg Depression Rating Scale (MADRS; (Montgomery and Åsberg, 1979); range in the whole sample, 0–46) and the self-reported revised Beck's Depression Inventory (BDI-II; (Beck et al., 1996); range in the whole sample, 0–53; 1 missing report).

History of childhood abuse was measured via the short form of the Childhood Trauma Questionnaire (CTQ-SF (Wingenfeld et al., 2010);). The CTQ-SF is a self-reported retrospective questionnaire that assesses five types of adverse childhood experiences, and each scale consists of five items rated with a five-point Likert scale ranging from 1 (never true) to 5 (very often true), and severity scores range from 5 to 25. Good reliability, validity, and item consistency have been demonstrated for the German version of the CTQ-SF (Klinitzke et al., 2012). In the current study *childhood abuse* comprised subscales emotional, physical and sexual abuse (range in the SA group, 15–54).

Number of lifetime SAs was measured with a clinical interview done by a trained psychologist (LB and GW). The groups were split as *multiple* (n = 13; 59 %) and *single* SA (n = 9; 41 %).

Severity of suicide intent was measured with the total score of the Beck's Suicide Intent Scale (SIS; (Beck et al., 1974); range in the SA group, 8–26). The SIS measures both objective (time, suicide note, …) and subjective intent (attitude towards dying, …). While suicide intent is closely related to suicide attempt, it is a separate cognitive category that marks individual's desire to end their life (Hasley et al., 2008), and the severity of intent can vary substantially among individuals with a history of suicide attempt.

Impulsive suicide attempt was measured with the SIS item #15 “Degree of Premeditation”. Groups were formed as *impulsive* SA that comprised participants that answered “0. None” or “1. Suicide contemplated for 3 h or less prior to attempt” (n = 10; 45 %) and *non-impulsive* SA that comprised participants that answered “2. Suicide contemplated for more than 3 h prior to attempt” (n = 12; 55 %).

### 2.4 Data analysis

The three groups were compared on demographic and clinical variables with one-way analysis of variance (ANOVA) and chi-square tests.

Primary analysis. We aimed to measure differences between the stress hormones and groups across the two hair segments, approximately measuring time after the SA, i.e., peri-suicidal (1st centimeter, approximately 1 month before the sampling, closest to the scalp) and the next segment, which denotes time preceding suicide attempt (2nd centimeter, approximately 2 months before the sampling). Hair stress hormones were logarithmic-transformed and values over ±3 SD were excluded from the analyses (1 outlier for the cortisol-log and 2 outliers for the DHEA-log). We applied a mixed model and hair stress hormones were analyzed as the dependent while hair segment (1st or 2nd cm, *i.e.*, time) and group (SA, CC, HC) as independent fixed variables. Following results from a meta-analysis (Stalder et al., 2017), age, gender and hair weight were considered as covariates of no interest. Participants were considered as a random effect and random intercept was estimated. We ran an interaction model and investigated the main and interaction effects of the group with the hair segment (time). The alpha value was set to p < .05. Mixed models were estimated using the analysis of variance (type III) with Satterthwaite approximation for degrees of freedom and the parameters were estimated using maximum likelihood. Normality of the variance was checked via residual plots. If a main or an interaction effect were significant, we followed up with simpler models within a hair segment and across groups (ANOVA) or within a group and across hair segments (LMM). Significant results were also tested for sensitivity with covariates of no interest, depression severity, education, and time between sampling and analysis, one at a time. For any significant model, to measure the achieved power for detecting the main effects of group/hair segment, and/or interaction effect in the LMMs, we ran a parametric bootstrap simulation using 1000 iterations along different sample sizes (n = 50–250).Mixed models (with diagnostics and plotting) were run with ‘lme4’, ‘lmerTest’, ‘sjstats’, ‘multilevelTools’, ‘effectsize’ and ‘car’, achieved power with ‘simr’, while graphs were created with ‘ggplot2’, ‘wesanderson’ and ‘effects’ packages in R (V4.1.3). Supplementary Table S1 shows the number of excluded participants for the primary models and the descriptive hormone values.

Secondary analysis. To assess the effects of specific SA-related factors on hair stress hormones in the SA group, secondary analyses of variance were run with a corrected alpha value at p < .012. We calculated the mean value of the stress hormones across segments, which were then logarithmic transformed. Linear models tested the main effect of childhood abuse and severity of suicide intent (continuous variables), *multiple* versus *single* SA, and *impulsive* versus *non-impulsive* SA (nominal variables). In all secondary models, age, gender, and mean hair weight were considered as covariates of no interest. Significant models were tested for sensitivity with covariates of no interest, depression severity, education, time since last SA, and time between sampling and analysis, one at a time. In addition, multivariate sensitivity analyses were conducted, grouping variables of interest together with socio-demographic, clinical and measurement variables. It should be noted that, due to the limited number of participants in the SA group, these analyses are considered preliminary. Linear models (with diagnostics and plotting) were run with ‘car’, achieved power with ‘sjstats, while graphs were created with ‘ggplot2’, and ‘wesanderson’ packages in R (V4.1.3).

Supplementary Table S2 shows details for the secondary analyses.

## 3 Results

There were no differences among groups for age, gender or body mass index. The final sample consisted entirely of participants of White/Caucasian ethnicity. There were differences in education (SA vs HC group, p = .009), depression severity (both MADRS and BDI-II, SA and CC vs HC, p's < .001) and time from sampling to analysis (CC vs SA and HC, p's < .03; Table 1).

**Table 1:** Demographic and clinical variables in the three groups.

	SA (n = 22)	CC (n = 31)	HC (n = 22)	
Age (mean [SD])	29.5 [10.2]	30.0 [11.5]	30.5 [8.6]	F(2,72) = .05, p = .9
Gender (n [%])	16 [73 %]	21 [68 %]	12 [54 %]	Χ^2^(2) = 1.7, p = .4
BMI (mean [SD])^a^	25.3 [7.0]	25.1 [6.5]	24.4 [4.3]	F(2,70) = .14, p = .9
Education (n [%])^b^				
Primary education	1 [4 %]	1 [3 %]	0 [0 %]	Χ^2^(8) = 15.9, p = .04^f^
Secondary education	11 [50 %]	8 [26 %]	2 [9 %]	
Vocational education	0 [0 %]	4 [13 %]	3 [14 %]	
High school	6 [27 %]	11 [35 %]	8 [36 %]	
University	3 [14 %]	4 [13 %]	9 [41 %]	
Primary diagnosis (n [%])		31 [100 %]	/	
MDD	21 [95 %]			
PTSD	1 [5 %]			
MADRS (mean [SD])	23.8 [10.7]	22.1 [9.3]	1.7 [2.2]	F(2,72) = 48.7, p < .001^g^
BDI-II (mean [SD])^c^	29.1 [14.7]	22.2 [14.4]	3.1 [4.7]	F(2,71) = 26.0, p < .001^g^
Time to analysis (mean [SD])^d^	300 [171]	420 [169]	287 [143]	F(2,69) = 5.2, p = .008^h^
Time since SA (mean [SD])	16.8 [10.1]	/	/	
Number of previous SA (mean [SD])^e^	2.4 [5.2]	/	/	

Abbreviations: BDI-II= Beck's Depression Inventory; BMI= Body mass index; CC = clinical controls group; HC = healthy controls group; MADRS= Montgomery-Åsberg Depression Rating Scale; MDD = Major depressive disorder; PTSD= Post-traumatic stress disorder; SA = suicide attempt group; SD = standard deviation.

a Two participants in the CC group are missing information and two participants were detected as outliers.

b One participant in the SA and three participants in the CC group are missing information.

c One participant in the CC group did not fill out the questionnaire.

d Time between hair sampling and hair stress hormone analysis; three participants in the CC group are missing information.

e One participant was detected as outlier.

f Post-hoc comparisons showed differences between SA and HC groups (Χ^2^(4) = 13.5, p = .009).

g Post-hoc Tukey's test indicated significant difference between CC and HC (p < .001), and SA and HC (p < .001).

h Post-hoc comparisons showed differences between SA and CC group (p = .03) and CC and HC groups (p = .01).

### 3.1 Hair stress hormones across groups

There was no significant interaction effect for the cortisol-log values (F(2,55.0) = 2.3, p = .11, η^2^(partial) = .04 - small effect size; Fig. 1A).

**Fig. 1:**
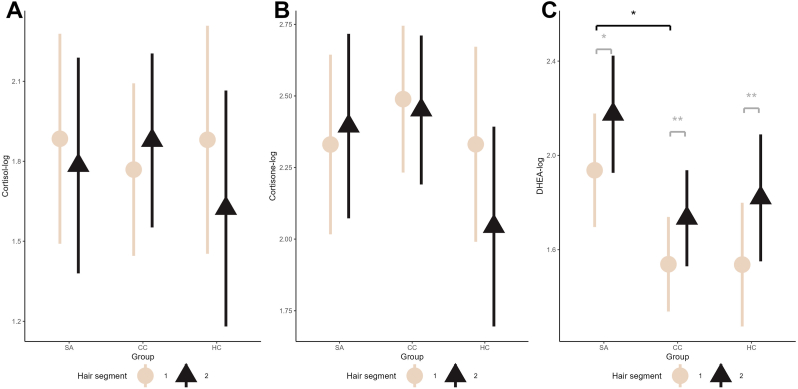
Linear mixed model estimates of (A) cortisol-log, (B) cortisone-log and (C) DHEA-log for each group and hair segment, corrected for age, gender and hair mass. Abbreviations: HC = healthy controls group; CC = clinical controls group; DHEA = dehydroepiandrosterone; -log = logarithmic transformed variable; SA = suicide attempt group *Note:* There was a marginal interaction effect for the cortisone-log (p = .07). Since it is below p < .05 threshold, we did not mark post-hoc tests in the graph. You can read the full results in the supplemental material. For DHEA-log following the main effect for hair segment (p < .001), post-hoc tests showed differences in each group (gray brackets), while for the main effect of group (p = .02) post-hoc tests showed significant differences among SA and CC for segment 1 (p = .01, black bracket) and marginally significant differences among SA and HC (p = .08).

There was a marginal interaction effect between group and hair segment cortisone-log (F(2, 59.3) = 2.8, p = .07, η^2^(partial) = .06 - medium ES). For cortisone-log there were group differences for hair segment 2 (further away in time) (hair segment 1, p = .6; hair segment 2, p = .05) with HC group having lowest values (SA vs CC p_corr_ = .9, SA vs HC p_corr_ = .2, CC vs HC p_corr_ = .09). There were also differences among hair segments in the HC group (SA, p = .6; CC, p = .5; HC, p = .01) with hair segment 1 having higher value. However, these results should be interpreted with caution, as the interaction was marginally significant (Fig. 1B).

There was no interaction effect for DHEA-log (F(2, 60.9) = .3, p = .7, η^2^(partial) < .001- trivial ES). There were however main effects of group (F(2, 63.6) = 4.3, p = .02, η^2^(partial) = .09- medium ES) and hair segment (F(1, 60.2) = 23.6, p < .001, η^2^(partial) = .27- large ES). Post-hoc analysis indicated that in hair segment 1 (time around the SA) there was a main effect of group (p = .01) with SA group having higher levels compared to CC and marginally higher than HC (SA vs CC p_corr_ = .01, SA vs HC p_corr_ = .08 and CC vs HC p_corr_ = .8). This effect was not observed for hair segment 2 (further away in time) (p = .1). All groups showed higher levels of DHEA-log in hair segment 2 (further away in time) compared to hair segment 1 (SA p = .02; CC p = .008, and HC p = .007) (Fig. 1C). Sensitivity analysis with additional covariates showed similar results. Results are listed in Supplementary Table S3A–C with notes on the sensitivity analyses.

The simulation of achieved power showed that the achieved power for the main effect of group and hair segment effects for the DHEA-log was around β = .75 and β = .95 respectively (Supplementary Fig. 1).

### 3.2 Effects of childhood abuse, multiple SA, severity of suicide intent and impulsiveness

**Fig. 2:**
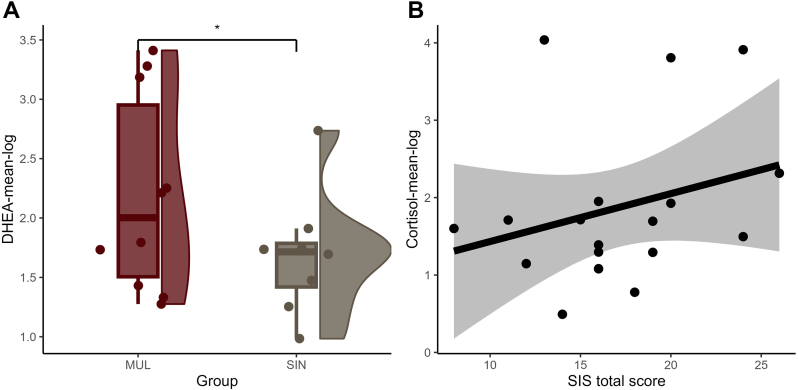
A Difference in the mean DHEA-log between participants who report multiple versus single suicide attempts (p_uncorr_ = .05); **2B** Association among the mean cortisol-log and level of suicidal intent for the last suicide attempt measured with the Beck Suicide Intent Scale (p_uncorr_ = .02). Abbreviations: DHEA = dehydroepiandrosterone; -log = logarithmic transformed variable; MUL = participants with multiple past suicide attempts; SIN = participants with a single past suicide attempt; SIS= Suicide intent scale.

Multiple SA group had higher levels of mean DHEA-log compared to group with persons with single suicide attempt on an uncorrected threshold (F(1,13) = 4.7, p = .05, η^2^(partial) = .26 - large ES; Fig. 2A). Sensitivity analysis with additional covariates yielded similar results on an uncorrected marginal level (p's < .1). Multivariate analyses showed that the addition of clinical or measurement covariates led to non-significant results (p > .05).

Moreover, severity of suicide intent was positively associated with mean cortisol-log (F(1,13) = 7.1, p = .02, η^2^(partial) = .35 - large ES; Fig. 2B) and mean cortisone-log (F(1,13) = 7.4, p = .02, η^2^(partial) = .36 - large ES), also on an uncorrected threshold. Sensitivity analyses with additional covariates showed similar results (p's < .05). Multivariate models were mostly non-significant (p > .05).

Childhood abuse and impulsive vs non-impulsive group did not show an association with any stress hormones.

Results are summarized in Supplementary Table S4A–C with notes on the sensitivity analyses. Multivariate analyses are presented in Supplementary Table S5A–C.

## 4 Discussion

This study tested the hypothesis that HPA axis dysregulation is associated with the history of *recent* SA. Specifically, we measured hair stress hormones in a well-defined SA group and compared to both CC and HC groups. We hypothesized that hair glucocorticoid and DHEA levels would be lower in the SA group in the peri-suicidal period, indicating inadequate HPA response to stressors surrounding attempt. Contrary to expectations, our main novel finding revealed significantly higher DHEA levels in segment 1, representing the month around the suicide attempt, in the SA group compared to CC group and marginally significant compared to the HC group, after correction for multiple comparisons. Furthermore, in the exploratory analyses, individuals with multiple SAs exhibited higher hair mean DHEA levels than individuals with single suicide attempt. Glucocorticoid analysis showed only marginal interaction effects, primarily driven by the HC group. However, mean levels of glucocorticoid were associated with higher suicide intent in the most recent SA. No measures were associated with childhood abuse or impulsiveness.

Only a few studies previously investigated DHEA levels in SA and showed mixed results. Higher blood serum DHEA was observed in male veterans with a lifetime history of SA compared to those without any history (Butterfield et al., 2005). Moreover, sulphate ester DHEA (DHEA-S) measured in the cerebrospinal fluid was higher in men but not women with a lifetime history of SA (Chatzittofis et al., 2013). In contrasts, one study reported lower levels of plasma DHEA and DHEA-S in individuals with SA history and a negative association with suicide ideation in veterans with combat exposure (Sher et al., 2018). Our study suggests that individuals with SA history, especially those with multiple attempts, have higher levels of DHEA. DHEA is often considered as a glucocorticoid antagonist in the stress response system (Kalimi et al., 1994; Kamin and Kertes, 2017) and it mediates both short and long term stress responses (Dutheil et al., 2021). In that context, our findings may seem counterintuitive. We may suggest that increased levels of DHEA reflect a disruption in DHEA metabolism, altered adrenal metabolism or dysregulated feedback in cortisol and DHEA (Ahmed et al., 2023). Evidence from animal studies suggests that unlike cortisol, which shows adaptability over repeated stress exposure, DHEA levels increase in a sustained manner (Maninger et al., 2010). Altered DHEA levels may also have implications for the brain functioning as it is connected to multiple neurotransmitter systems (Kamin and Kertes, 2017). For example, the γ-aminobutyric acid (GABA) system is directly modulated by the neurosteroids (Pérez-Neri et al., 2008). Post-mortem studies showed alterations in gene or protein expression in several components of GABA system in individuals who died by suicide (Pabba and Sibille, 2016). An additional target for the DHEA is the glutamatergic N-methyl-D-aspartate (NMDA) receptor (Zoupa et al., 2019). It is thus conceivable that the DHEA might be increasing suicidal risk via the NMDA receptor binding. Supporting this speculation, a pharmacological study showed that ketamine, an NMDA-receptor antagonist is efficient in reducing suicidal ideation (Jollant et al., 2023).

While alterations in cortisol levels have been associated with chronic stress exposure (Russell et al., 2012), little is known about the relative impact of acute life events versus cumulative daily stressors on other long-term endocrine markers. Evidence from hair cortisol studies suggests that global, sustained stress rather than single stressful events contributes most strongly to elevated cortisol levels over time (Stetler and Guinn, 2020). However, comparable data for DHEA are lacking. Future studies should therefore examine whether long-term DHEA concentrations similarly reflect chronic allostatic load (Juster et al., 2010) or respond primarily to acute stressors.

Another important aspect may be the role of sex differences in both endocrine regulation and psychopathology. A recent meta-analysis reported that depressed females have higher hair cortisol concentrations compared to depressed males, suggesting greater cumulative cortisol exposure in women with depression (Wang et al., 2024). Although the present study did not permit stratified analyses due to the limited sample size and a higher proportion of female participants, we controlled for gender, age, and hair weight in all analyses. Beyond cortisol, sex-related differences in other stress hormones have also been reported. For instance, DHEA levels are typically higher in women than in men (Kroboth et al., 1999; Šulcová et al., 1997), whereas elevated serum DHEA levels have been observed in both male and female patients with MDD (Kurita et al., 2013). To date, however, no study has systematically examined sex-specific differences in hair DHEA concentrations among psychiatric populations. Epidemiological evidence further indicates that men and women differ in suicidal behavior, with men more often engaging in violent and lethal methods, while women report higher rates of suicide attempts (Barrigon and Cegla-Schvartzman, 2020; Bommersbach et al., 2022; Schrijvers et al., 2012). Taken together, it is plausible that these behavioral and biological sex differences associate to distinct hormone profiles and vulnerability pathways leading to suicidal crises. Future studies should aim to recruit larger, sex-balanced samples to examine these mechanisms in greater depth.

Our analysis also revealed that SA and CC had similar levels of hair cortisol and cortisone across hair segments compared to HC (Fig. 1A and B). Previous research indicated hypoactive baseline and reactive cortisol in individuals with past SAs (Keilp et al., 2016; Melhem et al., 2016; O'Connor et al., 2017), hyperactive baseline (Genis-Mendoza et al., 2022; Lewitzka et al., 2018) as well as no differences (Stanley et al., 2019). Underscoring mixed results, a recent meta-analysis of thirty studies on peripheral cortisol levels measured in blood and saliva in suicide behavior reported a significant heterogeneity of studies due to varying methodology (Hernández-Díaz et al., 2020), sampling time with regard to the most recent SA (Lewitzka et al., 2018) as well as potential clinical and neurobiological heterogeneity of SA individuals. In our study, we analyzed two segments that gave us an opportunity to compare the overall HPA axis activity in the period around and period before the act, covering a time interval of approximately 2 months. As both SA and CC had similar levels in cortisol and cortisone across hair segments, nominally higher than HC, who furthermore showed a difference across hair segments in cortisone, implying a possible adaptive functioning of HPA, as was recently shown in another study on post-partum depression (Sadeghi-Bahmani et al., 2025). We may consider that alterations in hair glucocorticoids may rather reflect the effects of diagnosis and could not be considered a specific marker of SA or SA transition.

Recent studies have evaluated the use of hair cortisol concentration as a general biomarker for stress-related mental health conditions; however, all point to heterogeneity in the findings. For MDD, a meta-analysis found no significant overall difference in hair cortisol between depressed individuals and healthy controls (Psarraki et al., 2021). The authors emphasized that these findings were highly heterogeneous and likely influenced by factors independent of depression, such as suicidal behavior. In contrast, other reviews highlighted hair cortisol potential utility: Mlili et al. (2023) focused on the perinatal context, suggesting that changes in cortisol during pregnancy and postpartum could reflect cumulative stress and potentially act as a non-invasive marker of perinatal depressive symptoms, though they cautioned that longitudinal evidence is still limited. Furthermore, there are mixed results regarding hair cortisol as a biomarker for treatment outcomes, with the notion that individuals with higher baseline hair cortisol were sometimes associated with a greater response to psychological interventions (Botschek et al., 2023). Lastly, a recent study on the general population and subclinical symptoms did not find an association between hair cortisol and those self-reported measures (Torrecilla and Barrantes-Vidal, 2021).

Our exploratory preliminary analysis of possible contributing factors to the levels of hair stress hormones showed that the frequency of previous attempts was associated with DHEA levels. Our (Lübbert et al., 2021) and other's (Choi et al., 2013) previous research showed that individuals with multiple attempts are younger and have more experiences of interpersonal conflicts. It is, therefore, appealing to hypothesize that higher levels of DHEA reflect multiple interpersonal crises that may lead to, or themselves reflect, the dysregulation of the stress response system and stress homeostasis.

Unlike (Herzog et al., 2023), who measured acute cortisol reactivity to Trier Social Stress Test (TSST) in suicide attempters (finding attenuated responses in high-intent subjects), we assessed chronic glucocorticoid activity via hair analysis and observed positive associations with suicide intent. Individuals exhibiting high suicidal intent during past attempts face elevated future suicide risk (Pierce, 1981), characterized by extensive planning, preparatory behaviors, and expectation of death. Thus, the present correlation may indicate that high-intent suicide attempters constitute a biologically distinct subgroup, possibly marked by cortisol hyperreactivity or stress-system exhaustion. Our results may be in line with the recent meta-analysis on the dexamethasone suppression test also suggests that, among other factors, discrepancies in findings between persons with history of SA and persons who died by suicide may be explained by differences in the level of suicidal intent. However, intent is not the sole predictor of death by suicide; even low-intent attempts may prove fatal due to situational factors (e.g., method accessibility, rescue potential).

Moreover, we did not observe any association between stress hormones and impulsiveness as suggested by others (Stanley et al., 2019). The discrepancy may be due to hormones measurement methods, questionnaires used, variations in criteria of low vs high intent or impulsive SA, as well as putatively changed introspection regarding the suicidal act (Rimkeviciene et al., 2015). Lastly, we did not observe any association among self-reported childhood abuse and hair stress hormones in the SA group, in contrast to the reported negative relationship with hair cortisol reported by (Melhem et al., 2017). However, as indicated in a meta-analysis, the association between hair cortisol and childhood abuse (or other types of maltreatment) is heterogeneous (Schär et al., 2022) and dependent on other moderators such as study population, timing of adversity, measurements, etc. (Khoury et al., 2019).

As noted in the introduction, dysregulations of the HPA axis intersect with other systems thereby increasing risk for suicidal crisis (Lengvenyte et al., 2021). Physiological factors such as inflammatory factors (Dickerson et al., 2017; Jahangard et al., 2018; Neupane et al., 2023) can interact with endocrine and neural factors increasing vulnerability. Furthermore, behavioral factors such as impaired cognition (Becker et al., 2018; Richard-Devantoy et al., 2013; Wagner et al., 2015), decision making (Guillaume et al., 2013), risk-taking (Abdoli et al., 2022), aggressiveness (Drachman et al., 2022), rumination (Dauvermann et al., 2023; O'Connor et al., 2021), or affective temperaments (Baldessarini et al., 2017) also intersect with endocrine markers and increase risk. However, to disentangle complex multis-system to HPA axis large samples are needed (Hostinar et al., 2023).

## 5 Limitations

Our groups of persons with SA and clinical controls were individuals with depressive disorders, while other studies may have included multiple diagnostic categories, which may further show specific associations with HPA axis (Malisiova et al., 2021). Moreover, the data should be viewed in the context of ‘history of SA’ rather than having predictive value. Although past SA is a strong risk-factor for future SA, there may be significant neurobiological differences among those who engage in SA prospectively and those who die by suicide as compared to those with history of SA. Although we tried to keep the time between the SA and hair sampling as similar as possible, the actual duration varied (4–38 days) because the period after an SA is difficult to clinically manage (Zortea et al., 2020). Therefore, the segment more proximal to the scalp likely reflects cumulative hormone levels of the peri-suicidal period because it includes time both before and at once after the SA, not just the post-event time. The exact temporal separation is therefore not possible due to variability in hair growth and sampling time. An improved design including an added segment collected at least four weeks post-attempt would allow clearer differentiation of hormonal dynamics before, peri- and after the event.

We controlled variables such as age, gender and hair weight and did sensitivity analysis for depression severity, education and days to analysis. However, we cannot exclude that other factors influence stress hormone levels, for example hair growth and diet. No specific exclusion criteria were applied for general medication or supplement use. Although participants with inflammatory or neurological diseases were excluded from the study, unreported use of glucocorticoids or DHEA-like supplements cannot be entirely ruled out. We suggest that future studies should note medications or supplements that may influence endocrine measures. Moreover, we did not assess the severity of self-reported stress prior or retrospectively after the last SA. Although some reviews suggest that the self-reported measures do not relate with stress hormones measured in hair (Malisiova et al., 2021; Stalder et al., 2017), assessing multiple domains of experienced stress longitudinally may provide a better picture on the functionality of stress response system prior to a SA. Ideally, in addition to measuring hair stress hormones, studies should also collect daily saliva samples to assess dynamics of secretion and total output.

The secondary exploratory analyses, based on a limited sample size (n = 18), should be considered preliminary. The lack of significant findings in the multivariate regressions involving clinical or measurement variables likely stems from the low statistical power, which is a consequence of including multiple covariates in a small sample. Thus, these findings require validation in a larger, independent sample.

## 6 Conclusions

A novel finding is higher hair DHEA levels among individuals with a recent SA compared to individuals with a *current* depressive episode but no history of SA and controls with no history of mood disorder or SA. Higher mean DHEA was also observed in individuals with multiple lifetime SA. We speculate that it may indicate higher lifetime cumulative stress experiences and/or alterations in DHEA metabolism as a vulnerability marker. Our results contribute to the identification of biological stress markers in the context of suicidal risk measurement.

## Authors contributions

LC: Conceptualization; Formal analysis; Writing - Original Draft.

AKS: Investigation; Data Curation; Writing - Review & Editing.

LB: Investigation; Data Curation; Writing - Review & Editing.

AZ: Investigation; Data Curation; Writing - Review & Editing.

JW: Investigation; Data Curation; Writing - Review & Editing.

LMcC: Formal analysis; Writing - Review & Editing.

FP: Conceptualization; Writing - Review & Editing.

MA: Investigation; Writing - Review & Editing.

MW: Writing - Review & Editing.

FJ: Funding acquisition; Conceptualization; Investigation; Writing - Review & Editing.

GW: Funding acquisition; Conceptualization; Investigation; Data Curation; Supervision; Writing - Original Draft.

## Funding

This study was part of a linked standard research grant (“The choice of a violent suicidal means: a 10.13039/501100015668MRI study with computational modeling of decision-making”), from the 10.13039/100001455American Foundation for Suicide Prevention (10.13039/100001455AFSP) to F.J. and G.W., LSRG-1-086-19.

It was supported by the Interdisciplinary Center of Clinical Research of the Medical Faculty Jena (10.13039/100015683LC) and endorsed by the German Center for Mental 10.13039/100018696Health (10.13039/100015683LC, BB, MW).

We acknowledge support by the 10.13039/501100001659German Research Foundation Projekt-Nr. 512648189 and the Open Access Publication Fund of the Thueringer Universitaets-und Landesbibliothek Jena.

Funding sources had no role in the study design, data collection, analysis, and interpretation, writing of the report, and the decision to submit the article for publication.

## CRediT authorship contribution statement

**Lejla Colic:** Conceptualization, Formal analysis, Writing – original draft. **Anna Karoline Seiffert:** Data curation, Investigation, Writing – review & editing. **Lydia Bahlmann:** Data curation, Investigation, Writing – review & editing. **Ani Zerekidze:** Data curation, Investigation, Writing – review & editing. **Johanna Walther:** Data curation, Investigation, Writing – review & editing. **Larissa McClain:** Formal analysis, Writing – review & editing. **Bianca Besteher:** Writing – review & editing. **Fabricio Pereira:** Conceptualization, Writing – review & editing. **Mocrane Abbar:** Conceptualization, Writing – review & editing. **Martin Walter:** Writing – review & editing. **Fabrice Jollant:** Conceptualization, Funding acquisition, Investigation, Writing – review & editing. **Gerd Wagner:** Conceptualization, Data curation, Funding acquisition, Investigation, Supervision, Writing – original draft.

## Declaration of competing interest

MW is a member of the following advisory boards and gave presentations to the following companies: Bayer AG, Germany; Boehringer Ingelheim, Germany; and Biologische Heilmittel Heel GmbH, Germany. MW has further conducted studies with institutional research support from HEEL and Janssen Pharmaceutical Research for a clinical trial (IIT) on ketamine in patients with MDD, unrelated to this investigation.

All other authors report no biomedical financial interests or other potential conflicts of interest.

## Figures and Tables

**figs1:**
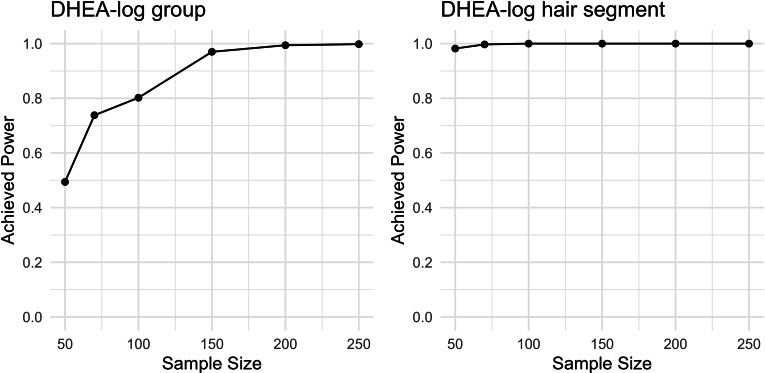


## Data Availability

Data will be made available on request.
