# Supplementary material for: Hair stress hormones in individuals with a recent suicide attempt experiencing a depressive episode
Source: Neurobiol Stress. 2025 Dec 2;40:100776. doi: 10.1016/j.ynstr.2025.100776 (PMC12721282; doi:10.1016/j.ynstr.2025.100776)
Supplement: Multimedia component 1 [file mmc1.docx]

# Supplementary Material

**Supplementary Table S1** Primary analysis. Details on the excluded participants and hair stress hormone values.

|  | SA group | CC group | HC group |
| --- | --- | --- | --- |
| Number of participants | 22 | 31 | 22 |
| Number of samples ^A^ | 44 | 61 | 44 |
| Excluded hair mass < 3 mg | 5 | 1 | 10 |
| **Number of samples in the final analysis** | **39** | **60** | **34** |
| Hair mass mg (mean [SD])^§^ | 6.4 [1.6] | 6.7 [1.2] | 6.4 [1.5] |
| Below detection values |  | 1 for cortisol |  |
|  |  |  |  |
| ***Raw values*** |  |  |  |
| Cortisol pg/mg (mean [SD])^B^ | 12.9 (19.2) | 8.3 (5.7) | 11.7 (24.9) |
| Cortisone pg/mg (mean [SD]) | 15.7 (17.2) | 14.9 (10.3) | 10.9 (9.5) |
| DHEA pg/mg (mean [SD]) | 10.4 (10.4) | 8.8 (16.5) | 6.6 (3.6) |
|  |  |  |  |
| ***Logarithmic transfor. values*** |  |  |  |
| Cortisol-log (mean [SD])^B^ | 1.9 (1.1) | 1.8 (0.8) | 1.7 (1.0) |
| Cortisone-log (mean [SD]) | 2.4 (0.8) | 2.5 (0.7) | 2.2 (0.6) |
| DHEA-log (mean [SD]) | 2.0 (0.8) | 1.7 (0.8) | 1.7 (0.5) |

Abbreviations: HC= healthy controls group; CC= clinical controls group; DHEA= dehydroepiandrosterone; -log= logarithmic transformed variable; SA= suicide attempt group; SD= standard deviation

^A^ One participant in the CC group did not provide sample for segment two

^B^ One participant in the CC group had cortisol below detection level thus sample is n= 59

^§^ Using linear mixed model, we tested if there are potential differences in hair mass between groups and segments. All main and interaction models were not significant (p’s> .12)

*Note*: the number of samples reflects two points for each group, *i.e.* the number of participants is SA n= 22, CC n= 31, and HC n= 22. Outliers were defined as values over $\pm$3 SD; there were 1 outlier in the HC group for the cortisol-log and 2 outliers in the CC group for the DHEA-log. For completeness, we present non-transformed raw values and logarithmically transformed values.

**Supplementary Table S2** Secondary analysis. Excluded participants, hair stress hormone values and clinical variables.

|  | SA group |
| --- | --- |
| Number of participants | 22 |
| Excluded hair mass < 3 mg in either segment^A^ | 4 |
| **Number of participants in the final analysis** | **18** |
| Hair mass mean mg (mean [SD]) mg | 6.6 [1.4] |
|  |  |
| ***Raw values*** |  |
| Cortisol pg/mg (mean [SD]) | 12.4 [17.8] |
| Cortisone pg/mg (mean [SD]) | 15.2 [17.0] |
| DHEA pg/mg (mean [SD]) | 9.6 [8.6] |
|  |  |
| ***Logarithmic transfor. values*** |  |
| Cortisol-log (mean [SD]) | 1.9 [1.0] |
| Cortisone-log (mean [SD]) | 2.4 [0.8] |
| DHEA-log (mean [SD]) | 2.0 [0.7] |
|  |  |
| ***Psychometric variables*** |  |
| Childhood abuse (mean [SD]) | 28.0 [10.3] |
| Participants with multiple suicide attempts (n [%]) | 10 [56%] |
| Severity of suicide intent (mean [SD]) | 17.1 [4.7] |
| Participants with impulsive suicide attempt (n [%]) | 10 [56%] |

Abbreviations: DHEA= dehydroepiandrosterone; -log= logarithmic-transformed variable; SA= suicide attempt group; SD= standard deviation

^A^ There were 1 participant in hair segment one, and 4 participants in hair segment 2 had < 3 mg hair weight with total exclusion of four participants

*Note*: Hormone values represent mean value across segments. Outliers were defined as values over $\pm$3 SD, and there were no outliers in any variable. For completeness, we present non-transformed raw values and logarithmically transformed values.

**Supplementary Table S3A**. Analysis of variance table for the group and hair segment on the cortisol-log.

| Fixed effects |  |  |  |  |  |  |
| --- | --- | --- | --- | --- | --- | --- |
|  | Sum Sq | Mean Sq | NumDF | DenDF | F value | Pr(>F) |
| Group | 0.01551 | 0.00776 | 2 | 59.749 | 0.0504 | 0.9508 |
| Hair segment | 0.19803 | 0.19803 | 1 | 54.324 | 1.2878 | 0.2614 |
| Age | 0.17006 | 0.17006 | 1 | 58.979 | 1.1059 | 0.2973 |
| Gender | 0.29853 | 0.29853 | 1 | 59.693 | 1.9413 | 0.1687 |
| Hair mass | 0.22556 | 0.22556 | 1 | 100.131 | 1.4668 | 0.2287 |
| **Group by Hair segment** | **0.69997** | **0.34998** | **2** | **55.045** | **2.2759** | **0.1123** |

Abbreviations: -log= logarithmic transformed variable

*Note*: Post-hoc results indicated there were no significant differences among groups in segments (Hair segment 1: F(2,63)= 0.03, p= .9; Hair segment 2: F(1,56)= 1.7, p= .2) or in each group among hair segments (SA: F= 0.4, p= .5; CC: F= 1.4, p= .2; HC: F= 2.5, p= .1). Results for the interaction effect remained similar with additional covariates (MADRS: p= .11; BDI-2: p= .07; education: p= .19; day to analysis: p= .16). There were no main effects of additional covariates (p’s> .36).

**Supplementary Table S3B**. Analysis of variance table for the group and hair segment on the cortisone-log.

| Fixed effects |  |  |  |  |  |  |
| --- | --- | --- | --- | --- | --- | --- |
|  | Sum Sq | Mean Sq | NumDF | DenDF | F value | Pr(>F) |
| Group | 0.18396 | 0.09198 | 2 | 62.244 | 0.9301 | 0.39994 |
| Hair segment | 0.22521 | 0.22521 | 1 | 58.544 | 2.2772 | 0.13666 |
| Age | 0.43578 | 0.43578 | 1 | 61.651 | 4.4064 | 0.03991 |
| Gender | 0.13392 | 0.13392 | 1 | 62.246 | 1.3541 | 0.24900 |
| Hair mass | 0.05387 | 0.05387 | 1 | 101.961 | 0.5447 | 0.46218 |
| **Group by Hair segment** | **0.55361** | **0.27681** | **2** | **59.339** | **2.7989** | **0.06893** |

Abbreviations: -log= logarithmic transformed variable

*Note*: Post-hoc results indicated there were significant differences among groups in hair segment 2 (further away in time) (Hair segment 1: F(2,64)= 0.4, p= .6; Hair segment 2: F(2,57)= 3.1, p= .05 [SA vs CC, p= .9; SA vs HC, p= .2; CC vs HC, p= .09) and in HC group among hair segments (SA: F= 0.2, p= .6; CC: F= 0.5, p= .5; HC: F= 8.1, p= .01). Results for the interaction effect remained similar with additional covariates (MADRS: p= .07; BDI-2: p= .07; education: p= .06; day to analysis: p= .07). There were no main effects of additional covariates (p’s> .67).

**Supplementary Table S3C**. Analysis of variance table for the group and hair segment on the DHEA-log.

| Fixed effects |  |  |  |  |  |  |
| --- | --- | --- | --- | --- | --- | --- |
|  | Sum Sq | Mean Sq | NumDF | DenDF | F value | Pr(>F) |
| **Group** | **0.61514** | **0.30757** | **2** | **63.575** | **4.3094** | **0.017575** |
| **Hair segment** | **1.68471** | **1.68471** | **1** | **60.182** | **23.6049** | **8.807e-06** |
| Age | 1.36699 | 1.36699 | 1 | 62.786 | 19.1531 | 4.647e-05 |
| Gender | 0.86532 | 0.86532 | 1 | 63.658 | 12.1242 | 0.000905 |
| Hair mass | 0.26627 | 0.26627 | 1 | 96.371 | 3.7308 | 0.056355 |
| **Group by Hair segment** | **0.04003** | **0.02001** | **2** | **60.957** | **0.2804** | **0.756441** |

Abbreviations: DHEA= dehydroepiandrosterone; -log= logarithmic transformed variable

*Note*: Post-hoc results indicated there were significant differences among groups in segment 1 (Hair segment 1: F(2,63)= 4.7 p= .012 [SA vs CC, p= .01; SA vs HC, p= .084; CC vs HC, p= .8]; Hair segment 2: F(1,56)= 2.2, p= .12 [SA vs CC, p= .12; SA vs HC, p= .7; CC vs HC, p= .5]). There were also differences among hair segments in groups (SA: F= 6.9, p= .017; CC: F= 8.1, p= .008; HC: F= 9.6, p= .007) where the hair segment 2 (further away from scalp) had higher levels of DHEA. Results for the main effect of group, hair segment and interaction respectively, remained similar with additional covariates (MADRS: p= .02/ <.001/ .76; BDI-2: p= .006/ <.001/ .69; education: p= .12/ <.001/ .73; day to analysis: p= .015/ <.001/ .75). There were no main effects of additional covariates (p’s> .10).

**Supplementary Table S4A**. Analysis of variance table for the exploratory analyses on the cortisol-log. For brevity, we noted statistics for the variables of interest, while models also included age, gender and hair weight.

| Tested variable | Sum Sq | Df | F value | Pr(>F) | η_p_^2^ |
| --- | --- | --- | --- | --- | --- |
| Childhood abuse | 0.46 | 1, 13 | 0.57 | .46 | 0.04 |
| Multiple vs single SA group | 0.09 | 1, 13 | 0.11 | .74 | 0.01 |
| **Severity of suicide intent^A^** | **3.86** | **1, 13** | **7.08** | **.020 *** | **0.35** |
| Impulsive vs non-impulsive SA group | 0.44 | 1, 13 | 0.54 | .47 | 0.04 |

Abbreviations: -log= logarithmic transformed variable; SA= suicide attempt group

^A^ Sensitivity analysis included adding depression severity (MADRS, BDI-II), education, time since last SA, and time between sampling and analysis, as covariates one at a time. The results remained similar, and models were in the range of p= .02-.05 with η_p_^2^= 0.29-0.45). There were no main effects of additional covariates (p’s> .3).

*Note*: Results are presented on an unadjusted statistical threshold and should be considered preliminary. Η_p_^2^= 0.35 represents large effect size.

**Supplementary Table S4B**. Analysis of variance table for the exploratory analyses on the cortisone-log. For brevity, we noted statistics for the variables of interest, while models also included age, gender and hair weight.

| Tested variable | Sum Sq | Df | F value | Pr(>F) | η_p_^2^ |
| --- | --- | --- | --- | --- | --- |
| Childhood abuse | 0.24 | 1, 13 | 0.44 | 0.52 | 0.03 |
| Multiple vs single SA group | 0.06 | 1, 13 | 0.12 | 0.74 | 0.01 |
| **Severity of suicide intent^B^** | **2.60** | **1, 13** | **7.39** | **0.017 *** | **0.36** |
| Impulsive vs non-impulsive SA group | 0.62 | 1, 13 | 1.24 | 0.28 | 0.09 |

Abbreviations: -log= logarithmic transformed variable; SA= suicide attempt group

^B^ Sensitivity analysis included adding depression severity (MADRS, BDI-II), education, time since last SA, and time between sampling and analysis, as covariates one at a time. The results remained similar, and models were in the range of p= .02-.04 with η_p_^2^= 0.32-0.48). There were no main effects of additional covariates (p’s> .4).

*Note*: Results are presented on an unadjusted statistical threshold and should be considered preliminary. Η_p_^2^= 0.36 represents large effect size.

**Supplementary Table S4C**. Analysis of variance table for the exploratory analyses on the DHEA-log. For brevity, we noted statistics for the variables of interest, while models also included age, gender and hair weight.

| Tested variable | Sum Sq | Df | F value | Pr(>F) | η_p_^2^ |
| --- | --- | --- | --- | --- | --- |
| Childhood abuse | 0.26 | 1, 13 | 0.47 | 0.50 | 0.03 |
| **Multiple vs single SA group^C^** | **1.96** | **1, 13** | **4.67** | **0.050 *** | **0.26** |
| Severity of suicide intent | 0.01 | 1, 13 | 0.01 | 0.92 | 0.001 |
| Impulsive vs non-impulsive SA group | 0.30 | 1, 13 | 0.54 | 0.47 | 0.04 |

Abbreviations: -log= logarithmic transformed variable; SA= suicide attempt group

^C^ Sensitivity analysis included adding depression severity (MADRS, BDI-II), education, time since last SA, and time between sampling and analysis, as covariates one at a time. The results remained similar, and models were in the range of p= .06-.09 with η_p_^2^= 0.23-0.28). There were no main effects of additional covariates (p’s> .5).

*Note*: Results are presented on an unadjusted statistical threshold and should be considered preliminary. Η_p_^2^= 0.26 represents large effect size.

**Table S5A-1** Analysis of variance table for the exploratory analyses on the mean cortisol-log. Models include variables of interest.

|  | Sum Sq | Df | F value | Pr(>F) |
| --- | --- | --- | --- | --- |
| Intercept | 2.1544 | 1,13 | 1.8704 | 0.1946 |
| Childhood abuse | 1.8082 | 1,13 | 1.5699 | 0.2323 |
| Multiple vs single SA group | 0.4212 | 1,13 | 0.3657 | 0.5558 |
| **Severity of suicide intent** | **1.2178** | **1,13** | **1.0573** | **0.3226** |
| Impulsive vs non-impulsive SA group | 0.0171 | 1,13 | 0.0149 | 0.9048 |

**Table S5A-2** Analysis of variance table for the exploratory analyses on the mean cortisol-log. Models include variable of interest and socio-demographic covariates.

|  | Sum Sq | Df | F value | Pr(>F) |
| --- | --- | --- | --- | --- |
| Intercept | 2.1860 | 1,10 | 2.5143 | 0.14390 |
| **Severity of suicide intent** | **4.9265** | **1,10** | **5.6663** | **0.03859** |
| Age | 4.0469 | 1,10 | 4.6546 | 0.05635 |
| Gender | 0.1761 | 1,10 | 0.2026 | 0.66223 |
| Education | 1.8954 | 3,10 | 0.7267 | 0.55897 |

**Table S5A-3** Analysis of variance table for the exploratory analyses on the mean cortisol-log. Models include variable of interest and clinical variables.

|  | Sum Sq | Df | F value | Pr(>F) |
| --- | --- | --- | --- | --- |
| Intercept | 1.0980 | 1,14 | 0.9401 | 0.3487 |
| **Severity of suicide intent** | **1.2585** | **1,14** | **1.0775** | **0.3169** |
| MADRS | 0.2588 | 1,14 | 0.2216 | 0.6451 |
| BDI-II | 0.0004 | 1,14 | 0.0003 | 0.9857 |

**Table S5A-4** Analysis of variance table for the exploratory analyses on the mean cortisol-log. Models include variable of interest and measurement covariates.

|  | Sum Sq | Df | F value | Pr(>F) |
| --- | --- | --- | --- | --- |
| Intercept | 4.5826 | 1,13 | 4.9220 | 0.04494 |
| **Severity of suicide intent** | **1.1603** | **1,13** | **1.2463** | **0.28448** |
| Hair weight (mean) | 4.0078 | 1,13 | 4.3046 | 0.05842 |
| Time between sampling and analysis | 1.1210 | 1,13 | 1.2040 | 0.29243 |
| Time since last SA | 0.0231 | 1,13 | 0.0248 | 0.87722 |

**Table S5B-1** Analysis of variance table for the exploratory analyses on the mean cortisone-log. Models include variables of interest.

|  | Sum Sq | Df | F value | Pr(>F) |
| --- | --- | --- | --- | --- |
| Intercept | 3.2676 | 1,13 | 5.3295 | 0.03805 |
| Childhood abuse | 0.9210 | 1,13 | 1.5021 | 0.24209 |
| Multiple vs single SA group | 0.2547 | 1,13 | 0.4154 | 0.53047 |
| **Severity of suicide intent** | **0.7144** | **1,13** | **1.1652** | **0.30001** |
| Impulsive vs non-impulsive SA group | 0.1094 | 1,13 | 0.1784 | 0.67967 |

**Table S5B-2** Analysis of variance table for the exploratory analyses on the mean cortisone-log. Models include variable of interest and socio-demographic covariates.

|  | Sum Sq | Df | F value | Pr(>F) |
| --- | --- | --- | --- | --- |
| Intercept | 0.3575 | 1,10 | 0.8612 | 0.37526 |
| **Severity of suicide intent** | **3.3338** | **1,10** | **8.0302** | **0.01774** |
| Age | 2.9853 | 1,10 | 7.1907 | 0.02303 |
| Gender | 0.0979 | 1,10 | 0.2358 | 0.63772 |
| Education | 1.1571 | 3,10 | 0.9291 | 0.46204 |

**Table S5B-3** Analysis of variance table for the exploratory analyses on the mean cortisone-log. Models include variable of interest and clinical variables.

|  | Sum Sq | Df | F value | Pr(>F) |
| --- | --- | --- | --- | --- |
| Intercept | 2.3410 | 1,14 | 3.7752 | 0.0724 |
| **Severity of suicide intent** | **1.0389** | **1,14** | **1.6755** | **0.2165** |
| MADRS | 0.1029 | 1,14 | 0.1659 | 0.6900 |
| BDI-II | 0.0230 | 1,14 | 0.0371 | 0.8500 |

**Table S5B-4** Analysis of variance table for the exploratory analyses on the mean cortsione-log. Models include variable of interest and measurement covariates.

|  | Sum Sq | Df | F value | Pr(>F) |
| --- | --- | --- | --- | --- |
| Intercept | 3.1911 | 1,13 | 5.4432 | 0.03635 |
| **Severity of suicide intent** | **1.1456** | **1,13** | **1.9541** | **0.18554** |
| Hair weight (mean) | 1.3178 | 1,13 | 2.2478 | 0.15769 |
| Time between sampling and analysis | 0.5057 | 1,13 | 0.8627 | 0.36992 |
| Time since last SA | 0.0098 | 1,13 | 0.0168 | 0.89895 |

**Table S5C-1** Analysis of variance table for the exploratory analyses on the mean DHEA-log. Models include variables of interest.

|  | Sum Sq | Df | F value | Pr(>F) |
| --- | --- | --- | --- | --- |
| Intercept | 3.7554 | 1, 13 | 8.7740 | 0.01101 |
| Childhood abuse | 1.2052 | 1, 13 | 2.8159 | 0.11720 |
| **Multiple vs single SA group** | **2.1484** | **1, 13** | **5.0195** | **0.04316** |
| Severity of suicide intent | 0.9655 | 1, 13 | 2.2557 | 0.15702 |
| Impulsive vs non-impulsive SA group | 1.1468 | 1, 13 | 2.6795 | 0.12562 |

**Table S5C-2** Analysis of variance table for the exploratory analyses on the mean DHEA-log. Models include variable of interest and socio-demographic covariates.

|  | Sum Sq | Df | F value | Pr(>F) |
| --- | --- | --- | --- | --- |
| Intercept | 11.6893 | 1,10 | 31.2518 | 0.0002308 |
| **Multiple vs single SA group** | **1.5184** | **1,10** | **4.0596** | **0.0715948** |
| Age | 0.1368 | 1,10 | 0.3657 | 0.5588356 |
| Gender | 1.7129 | 1,10 | 4.5795 | 0.0580369 |
| Education | 0.2005 | 3,10 | 0.1786 | 0.9084508 |

**Table S5C-3** Analysis of variance table for the exploratory analyses on the mean DHEA-log. Models include variable of interest and clinical variables.

|  | Sum Sq | Df | F value | Pr(>F) |
| --- | --- | --- | --- | --- |
| Intercept | 14.1128 | 1,14 | 25.3242 | 0.0001832 |
| **Multiple vs single SA group** | **0.8642** | **1,14** | **1.5508** | **0.2334625** |
| MADRS | **0.1725** | 1,14 | 0.3096 | 0.5867070 |
| BDI-II | 0.0000 | 1,14 | 0.0001 | 0.9932409 |

**Table S5C-4** Analysis of variance table for the exploratory analyses on the mean DHEA-log. Models include variable of interest and measurement covariates.

|  | Sum Sq | Df | F value | Pr(>F) |
| --- | --- | --- | --- | --- |
| Intercept | 0.8477 | 1,13 | 1.5407 | 0.2364 |
| **Multiple vs single SA group** | **0.8586** | **1,13** | **1.5605** | **0.2336** |
| Hair weight (mean) | 0.6878 | 1,13 | 1.2501 | 0.2838 |
| Time between sampling and analysis | 0.7088 | 1,13 | 1.2883 | 0.2769 |
| Time since last SA | 0.0810 | 1,13 | 0.1471 | 0.7075 |

Abbreviations: BDI-II= Beck’s Depression Inventory; DHEA= dehydroepiandrosterone; -log= logarithmic transformed variable; MADRS= Montgomery-Åsberg Depression Rating Scale; SA= suicide attempt group.

**Supplementary Figure S1.** Simulated achieved power for the main effect of group and hair segment for the DHEA-log across different sample sizes.

Abbreviations: DHEA= dehydroepiandrosterone; -log= logarithmic transformed variable.

*Note*: The simulation was done for each main effects model separately. For the primary analysis (linear mixed models) the number of participants was 70. For the DHEA-log and main effect of group, for β= 0.8 recommended sample size is n≈ 100.
